# Whole genome resequencing reveals genetic relationships and differences between three types of Hainan local pig breeds

**DOI:** 10.3389/fvets.2025.1544321

**Published:** 2025-05-30

**Authors:** Jiayu Yan, Jing Chen, Shidao Zhao, Jie Chen, Lifan Zhang

**Affiliations:** ^1^Sanya Institute of Nanjing Agricultural University, College of Animal Science and Technology, Nanjing Agricultural University, Nanjing, China; ^2^Dongfang City Comprehensive Administrative Law Enforcement Bureau, Dongfang, China; ^3^Dazhang Jiaxing Pig Farm, Dongfang, China

**Keywords:** Duntou, Wuzhishan, Wenchang, pig, resequencing, genetic structure, selection signal

## Abstract

**Introduction:**

Hainan Island, a landmass separated from the Chinese mainland, is characterized by a tropical monsoon maritime climate with ample sunshine and rainfall. It features low-lying plains along the coast and a central mountainous core. These topographical and ecological conditions in the tropics have shaped the unique genetic characteristics of local pig breeds in Hainan. To date, the similarities and differences in the genetic characteristics of different pig breeds, especially pig breeds from different geographical regions, remain unclear.

**Methods:**

Whole genome resequencing were applied to 25 Duntou (DT) pigs from the western, 17 Wuzhishan (WZS) pigs from the central, and 23 Wenchang (WC) pigs from the eastern part of Hainan Island. Among the different pig populations, genetic relationships were assessed through ADMIXTURE analysis, phylogenetic tree construction, and multidimensional scaling analysis. Subsequently, genetic differences were determined by identifying differentially selection regions (DSRs) and the candidate genes within these regions.

**Results:**

After quality control, 13,614,302 autosomal single nucleotide polymorphisms (SNPs) were used in the present study. The results revealed that DT and WZS pigs had closer genetic connections, whereas the genetic connection between WC and the other two pig breeds was relatively distant. Moreover, 1,174 selected genes from DSRs were identified between DT and WZS pigs, WZS and WC pigs, and DT and WC pigs, which included a number of important candidates associated with the growth properties of WZS pigs, and farrowing and fat deposition in WC pigs. The identified genes are able to serve as a scientific underpinning for the conservation and diversified utilization of these indigenous pig breeds.

**Discussion:**

This study provides a genome-wide view of the genetic structure of pig breeds from different geographical locations in Hainan. Our data may provide the molecular characteristics for the formation of these pig breeds, thus helping us better understand the origin and genetic differences of local pig breeds in Hainan.

## 1 Introduction

Hainan Island, located in southern China, has a unique genetic resource shaped by its isolated geography and hot and humid climate. The central region limits external interaction, with long-term isolation and tropical rainforest climate increasing inbreeding in local populations, impacting their production performance. In contrast, the low-lying coastal areas surrounding the island exhibit significant environmental differences between east and west. The western region, characterized by long hours of sunshine and low rainfall, has fostered pig populations with strong heat tolerance, while the eastern region’s rainy and humid conditions have fostered individuals with superior stress and disease resistance. The local pig breeds of Hainan can be categorized into three main types: Duntou (DT), Wuzhishan (WZS), and Hainan black pigs. The Hainan Black pigs consist of four subtypes, namely Lingao (LG), Ding’an (DA), Tunchang (TC), and Wenchang (WC) pigs. The DT, WZS, and Hainan black pigs were primarily distributed in the western, central, and eastern regions of Hainan Island, respectively. Importantly, each pig breed has unique traits. For example, DT pigs are a breed developed due to residents introducing pig breeds from areas such as Beihai in Guangxi, Zhanjiang in Guangdong, and Haiphong in Vietnam before 1949, followed by a long-term process of selective breeding. Renowned for their high birth litter weight, these pigs are uniquely suitable for being transformed into delicious roasted suckling pigs at 20–30 days old (1, 2); WZS pigs are indigenous to the Wuzhishan region, an area characterized by lofty mountains and thick forests, which has led to a high level of isolation from the outside. Likely originating from the domestication of local wild boars and subsequent inbreeding, they are frequently utilized as laboratory animals because of their small body size (1). WC pigs trace their domestication back around AD 1600–1700, originating from Gongguan pigs introduced by residents from the Guangdong coast. These pigs are notable for their relatively high intramuscular fat content (1). Therefore, revealing the genetic differences between these pig breeds is significant for studying the germplasm characteristics of local pig breeds on Hainan Island.

Previous studies have revealed the genetic relationships between multiple European pig breeds, including Duroc, Large White, Landrace, and Pietrain, using microsatellite markers (3). Compared to microsatellite markers, whole genome resequencing technology offers higher throughput for analyzing all possible genetic variations in an individual genome and has now been widely applied in genetic diversity, selection signatures, and genome-wide association studies (GWAS) of agricultural animals (4–6). In the past years, several research groups have conducted analyses on the genetic structure of Hainan pig breeds and the genomic differences between Hainan pigs and other pig breeds. For example, TC pigs boast higher genetic diversity than Duroc and Landrace pigs, and there are many genes related to growth and development, meat quality, energy metabolism, and reproductive traits in the selection signals between them (7); In comparison with European pig breeds such as Duroc, Landrace, and Large White pigs, Hainan pigs including WZS, TC, and DA pigs not only possess higher genetic diversity but also have selected genes predominantly concentrated on characteristics like immunity, adaptability, reproduction, meat quality, and heat resistance (8, 9); and compared with Baoshan pigs and Saba pigs from high-altitude regions, the selection signals of Hainan black pigs such as TC and DA pigs contain quantitative trait locus (QTL) regions associated with backfat thickness, average daily gain, and drip loss (10). It is clear that the core of the above-mentioned studies lies in revealing the differences in genetic diversity and genome between two groups of Hainan black pigs, including WZS, TC, and DA pigs, and European pig breeds as well as high-altitude pig breeds in China. In addition, the population structure of Hainan pig breeds, including TC, DA, and WZS pigs, was analyzed, and the results indicated that Hainan pig breeds generally share close genetic relationships (9). However, notable differences are seen in the production performance of pig breeds from different regions of Hainan. So far, the genetic relationship and difference of pig breeds from different types or areas of Hainan pig breeds are still limited. In particular, there are scarcely any relevant reports on DT and WC pigs. Additionally, differential selection regions (DSRs) are genomic regions with shared differential selection signals across breeds depending on the differences in allele frequency of SNP. To precisely identify the genomic differential regions and their associated genes among pig breeds from different regions in Hainan, we selected representative pig breeds, specifically DT, WZS, and WC pigs, from different areas of Hainan Island. Subsequent analyses entailed a comprehensive characterization of their genetic structure using pairwise *F_ST_*, ADMIXTURE clustering, neighbor-Joining phylogenetic tree, and multidimensional scaling analysis. Employing the *F_ST_* framework, we further identified differential selection regions (DSRs) and interrogated breed-specific candidate genes, thereby facilitating a deeper understanding of the relationships and genetic characteristics of local pig breeds across different regions of Hainan.

## 2 Materials and methods

### 2.1 Animal

A total of 65 pig individuals were used for genome resequencing. Among these, 25 DT individuals (11 males and 14 females) were sampled from Dazhang Jiaxing Pig Farm (Dongfang City, Hainan Province), which is located in the southwest of Hainan Island, a low-altitude plain area with the strongest sunlight and the least rainfall on the island; 23 WC individuals (10 males and 13 females) were sampled from Wenchang City, which lies in the northeast of Hainan Island, has a landscape dominated by coastal and low-hilly terraces and is counted among the island’s regions with the highest frequency of typhoons and the heaviest rainfall; and 17 WZS individuals (8 males and 9 females) were sampled from Wuzhishan City, which is sited in the central part of Hainan Island, is a mountainous region with high altitude, thick vegetation, and a high degree of natural seclusion, mainly characterized by a tropical mountain climate. All animal handling and collection procedures were approved by the Animal Ethics Committee of Nanjing Agricultural University. In addition, we downloaded from the EMBL-EBI^1^ the whole-genome resequencing data of six wild boars and their closely related species from Indonesia (accession number: PRJEB1683), which were utilized for population genetic analysis as outgroup population.

### 2.2 DNA extracting, library preparation, and sequencing

DNA was extracted from the ear or muscle tissues of pigs using the phenol-chloroform extraction method by Berry Genomics in Beijing, China. DNA quality was verified by monitoring 1% agarose gels and measuring with a Qubit DNA Assay kit using a Qubit 2.0 Fluorometer (Thermo Fisher Scientific, San Jose, CA, United States). Libraries for sequencing were constructed using a DNA library construction kit (Illumina, San Diego, CA, United States). Among these, 25 DT pigs were sequenced using the Illumina NovaSeq 6000 sequencing platform, whereas 23 WC and 17 WZS pigs were sequenced using the DNBSEQ-T7 sequencing platform (Berry Genomics, Beijing, China).

### 2.3 Quality control, genome mapping, and SNP calling

These following type of raw reads were removed: (i) reads with 3 nt unidentified nucleotides; (ii) reads contain the adaptor; (iii) reads with ≥20% bases having phred quality ≤5. Clean reads were aligned to the pig reference genome (*Sus scrofa* 11.1) using BWA v0.7.17 (11). Samtools v1.9 (12) and Picard tools v2.10.7 were used to sort the BAM file and duplicate markings for computation of the sequence depth of the whole genome, as well as the coverage and depth of each chromosome. SNPs were independently called from the raw data of Illumina NovaSeq 6000 and DNBSEQ-T7 sequencing platforms with GATK v4.2.5.0 (13–15). Then, the gVCF files were merged by GATK v4.2.5.0 and annotated by ANNOVAR (16). This integrated approach ensured robust variant detection while accounting for platform-specific biases.

The vcf file was filtered using vcftools v0.1.15 (17) to remove SNPs with more than two alleles. Next, PLINK v1.90 (18) was used to remove all SNPs with a missing rate greater than 10% or a minor allele frequency (MAF) less than 0.1 with the parameters “--geno 0.1 --maf 0.1.” Only SNPs located on autosomes were retained for further analysis. Moreover, the parameter “--hwe 0.01” was applied to exclude SNPs that deviated from Hardy–Weinberg equilibrium within each breed population.

### 2.4 Population genetic structure analysis

Pairwise genetic distances among individuals were estimated using the identity-by-state (IBS). Specifically, the genetic distance between two samples was computed following the method used in a previous study (19): *Dst* = (*IBS*2 + 0.5 × *IBS*1)/N. Here, *Dst* represents the average proportion of alleles shared, *IBS*1 and *IBS*2, respectively, denote the number of IBS loci that two individuals share at one or two alleles, and N is the total number of SNPs. The genetic distance between all pairwise combinations of individuals is given by 1-*Dst*. A pairwise genetic distance matrix, which encompassed the 1-*Dst* values for all sample pairs, was constructed using PLINK v1.90 with the command “--distance 1-ibs.” This distance matrix was utilized as the input for two subsequent analyses: (1) a neighbor-Joining phylogenetic tree was constructed using MEGA 11 to visualize the individual relationships (20); (2) multidimensional scaling (MDS) based on IBS, denoted as IBS-MDS, was carried out using the cmdscale function in R v4.3.3 (21). This function transformed the 1-*Dst* distance matrix into low-dimensional Euclidean coordinates. Consequently, it enabled the visualization of genetic distances among samples in an IBS-MDS plot, which was also generated using R v4.3.3. ADMIXTURE v1.3 (22) was used to determine the population structure by conducting four independent analyses with K ranging from 3 to 5 and the cross-validation (CV) error was calculated using the “--cv” command. Subsequently, the outcomes were graphically presented using R 4.3.3. To evaluate the overall genetic differentiation among populations, pairwise Weir-Cockerham *F_ST_* estimates (23) were computed using the --fst command in PLINK v1.90.

### 2.5 Identification of differentially selected regions and candidate genes

The DSRs were identified using the following criteria: (1) Fisher’s exact test and Bonferroni correction. Fisher’s exact test between each pair of breeds was performed using the “--fisher” command in PLINK v1.90, followed by Bonferroni correction; (2) *F*_ST_ calculation. R v4.3.3 were used to estimate the *F_ST_* value for each SNP between each pair of breeds based on the model proposed by Nicholson et al. (24) and Flori et al. (25); (3) Selection of differentially selected regions (DSRs). Firstly, Bonferroni correction was applied to calculate the corrected Fisher’s *p*-value. Secondly, SNPs with the 0.01% highest *F_ST_* value and corrected *p* value < 0.05 were considered extremely significant SNPs. Thirdly, SNPs with the 0.5% highest *F_ST_* value and corrected *p*-value < 0.05 were considered significant SNPs. Fourthly, starting from every extremely significant SNPs, we searched for both directions until two consecutive nonsignificant SNPs were encountered. Finally, any regions containing more than five significant SNPs without interruption by two or more consecutive nonsignificant SNPs were also encountered. (4) Visualization of *F_ST_* results. The R package of qqman 0.1.9 was used to visualize the *F_ST_* results (26). (5) Identification of candidate genes. The DSRs were compared to the pig reference genome version 109^2^ to identify genes within these regions. The gene list was also compared with the pigQTLdb database version 52 to obtain further functional information (27) and with the PigBiobank (28) to assess functional significance of candidate genes.

## 3 Results

### 3.1 Genome data quality metrics

An average of 191,169,667 raw reads and 28,675,450,085 raw bases were obtained from the 65 individuals. After quality control, an average of 187,388,444 clean reads and 28,108,266,572 clean bases were obtained (Supplementary Table S1). The average sequence depth, clean Q30, and clean GC content were 10.59, 90.06, and 43.14%, respectively. Moreover, an average mapping rate of 97.24% was obtained using the pig reference genome.

### 3.2 Identification of SNPs and quality control

A total of 45,220,534 SNPs across 65 samples were identified in the three pig breeds. During the filtering process, a total of 6,958,211 SNPs with more than one mutant genotype were removed, 3,495,402 SNPs were excluded as they were located on sex chromosomes and not used for further analysis, 3,542,624 SNPs were removed due to a missing rate of more than 0.10, 14,602,046 SNPs were excluded due to an MAF below 0.1, and 3,007,949 SNPs were removed for not being in line with the Hardy–Weinberg equilibrium. After discarding these SNPs, a final dataset of 13,614,302 SNPs was used for further analyses.

### 3.3 Genetic relationship among DT, WZS, and WC pig breeds

**Figure 1:**
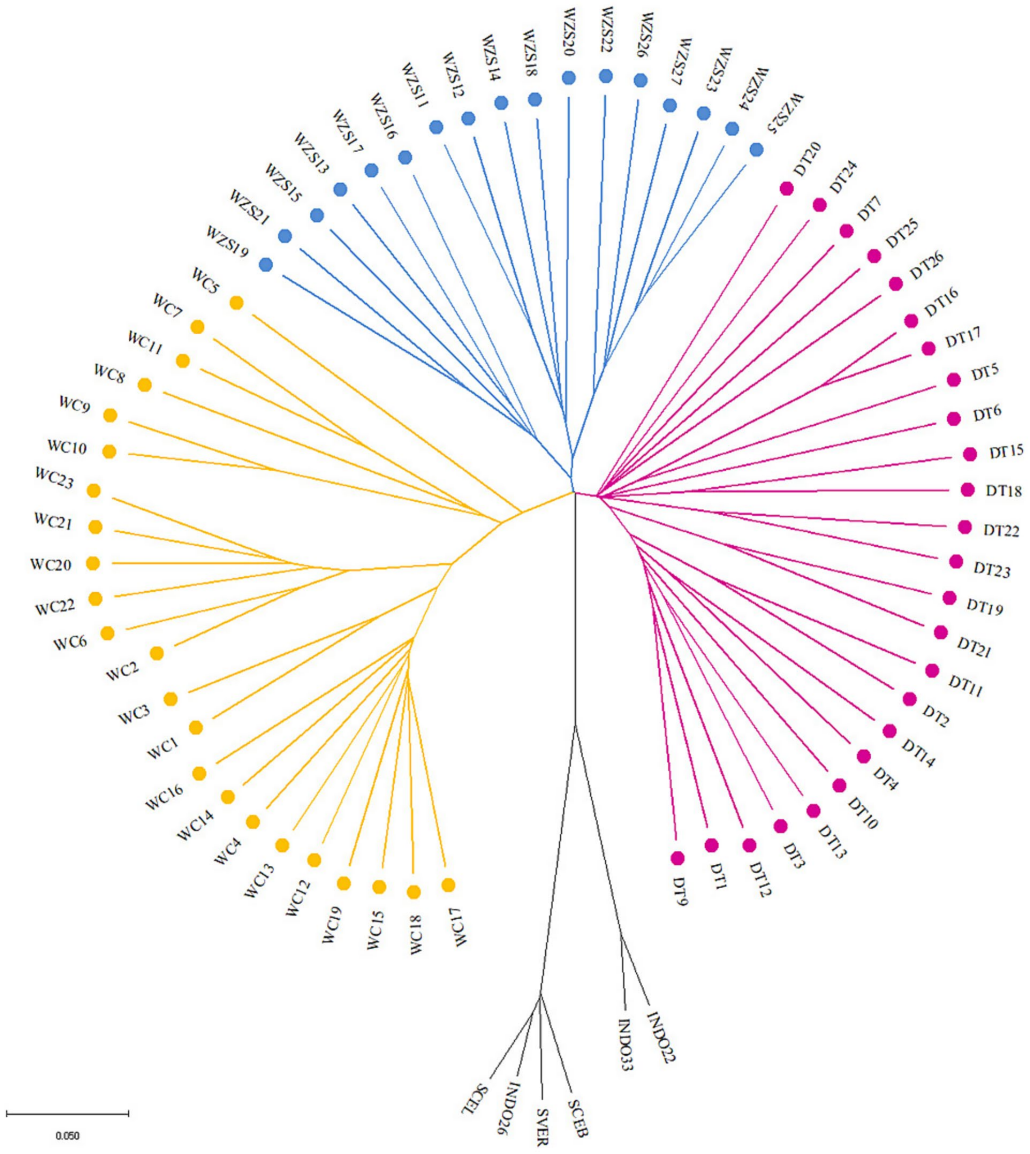
Neighbor-joining tree of 71 individuals among outgroup, DT, WZS, and WC pig breeds.

**Figure 2:**
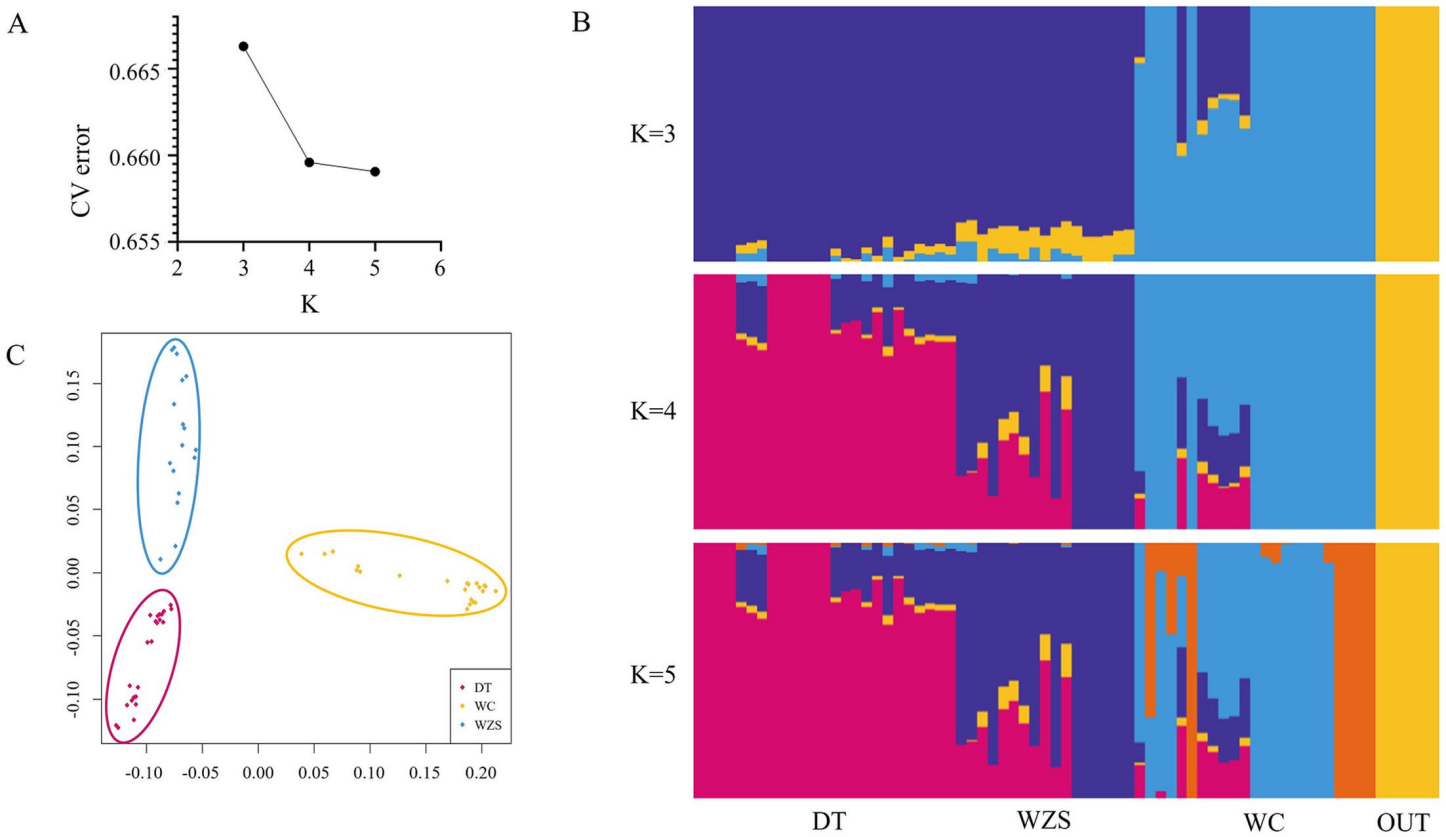
Population structure analysis. **(A,B)** Population structure of DunTou (DT), WuZhiShan (WZS), WenChang (WC) and outgroup (OUT) pig breeds. **(C)** IBS multidimensional scaling plots among DT, WZS, WC pig breeds.

To understand the population relationships among the three Hainan pig breeds, we constructed a neighbor-joining tree of the outgroup, DT, WZS, and WC pig breeds at the individual level (Figure 1), which indicated that the 71 samples could be clearly divided into four distinct clusters. DT and WZS pigs were located on the same major branch, whereas the outgroup and WC pigs, respectively, constituted distinct branches. A similar pattern was observed in the ADMIXTURE analysis, which revealed that WC pigs were distinguishable from the other two breeds at K = 3 (Figure 2B). Furthermore, K = 4 was identified as the optimal number of populations, which showed that all four groups were differentiated (Figures 2A,B).

Furthermore, an IBS plot was constructed to clarify the genetic relationships among these three pig breeds at the population level, which also showed that the spatial distance between the DT and WZS pigs was shorter (Figure 2C). Additionally, the weighted Weir and Cockerham *F_ST_* value between DT and WZS pigs was 0.09, between DT and WC pigs, was 0.17, and between WC and WZS pigs was 0.17, which again indicated that DT and WZS pigs showed notably shorter genetic distances.

### 3.4 DSRs and candidate genes in DT, WZS, and WC pig breeds

**Figure 3:**
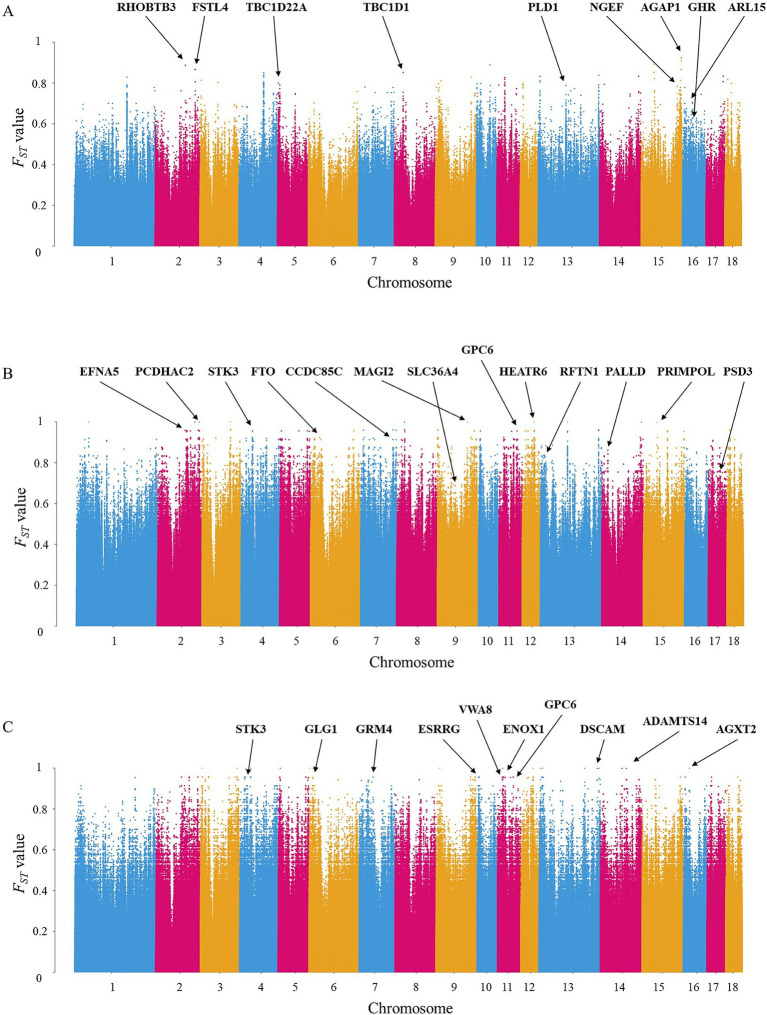
Manhattan plot of *F_ST_* value among three Hainan local pig breeds. **(A)** Manhattan plot between DT and WZS pigs. SNPs with the top 0.01% *F_ST_* value (0.6270) were considered to be extremely significantly different between the two breeds, and SNPs with the top 0.5% *F_ST_* value (0.3357) were considered to be significantly different between the two breeds. **(B)** Manhattan plot between DT and WC pigs. SNPs with the top 0.01% *F_ST_* value (0.8334) were considered to be extremely significantly different between the two breeds, and SNPs with the top 0.5% *F_ST_* value (0.5503) were considered to be significantly different between the two breeds. **(C)** Manhattan plot between WC and WZS pigs. SNPs in the top 0.01% *F_ST_* (0.8445) were considered extremely significant, and those in the top 0.5% *F_ST_* (0.5493) were considered significantly different between the two breeds.

Between DT and WZS pigs, 1,349 DSRs were mapped to 292 unique genes (Supplementary Table S2). Several genes related to growth traitsnamely *GHR*, *ARL15*, and *PLD1* (Table 1 and Figure 3A), were identified. Moreover, genes harboring SNPs with extremely high *F_ST_* values, such as *RHOBTB3*, *FSTL4*, and *AGAP1*, were pinpointed.

**Table 1:** Candidate genes associated with important traits between DT and WZS.

Gene	Chr	DSRs	Number of SNPs	Top 0.5%	TOP 0.01%	Function
*ARL15*	16	DSR1251-1252	9	6	5	Loin muscle area (28); Pig’s growth (33)
*GHR*	16	DSR1242	14	11	0	Animal growth (30–32)
*NGEF*	15	DSR1165-1170	63	46	9	ADG (28); Feed efficiency and growth rate in pigs (49)
*PLD1*	13	DSR949-950	12	11	5	Growth traits in pigs (50)
*TBC1D1*	8	DSR561-570	90	72	7	Teat number (28); Obesity and production traits (51)
*TBC1D22A*	5	DSR402-409	57	44	9	ADG (28); Feed efficiency and fatness traits in pigs (52)

ARL15, ADP ribosylation factor, like GTPase 15; GHR, growth hormone receptor; NGEF, Neuronal guanine nucleotide exchange factor; PLD1,: Phospholipase D1; TBC1D1, TBC1 domain family member 1; TBC1D22A, TBC1 domain family member 22A; ADG, average daily again.

Between DT and WC pigs, 3,638 DSRs were mapped to 585 unique genes (Supplementary Table S3). Our analysis further revealed that specific genes were associated with various traits: *SLC36A4* and *PSD3* were linked to litter weight, *FTO* was implicated in fat development, and *EFNA5* and *RFTN1* were associated with meat quality (Table 2 and Figure 3B). Moreover, genes harboring SNPs with extremely high *F_ST_* values, such as *MAGI2*, *HEATR6*, and *PRIMPOL*, were detected.

**Table 2:** Candidate genes associated with important traits between DT and WC.

Gene	Chr	DSRs	Number of SNPs	Top 0.5%	Top 0.01%	Function
*CCDC85C*	7	DSR1742	8	4	3	Granulosa cell proliferation in chickens (53)
*EFNA5*	2	DSR358	4	2	2	Total litter weight born alive (28); Meat color in pigs (47)
*FTO*	6	DSR1282-1285	23	18	4	Fat deposition in various animals (54)
*GPC6*	11	DSR2642-2647	30	26	3	Skeletal growth in mice (35); Intramuscular fat in pigs (55)
*PALLD*	14	DSR3137-3140	38	34	1	Teat number (28); backfat thickness (28); Ovarian follicular atresia in pigs (56)
*RFTN1*	13	DSR2899-2904	41	36	1	Meat color in pig (48)
*STK3*	4	DSR721-724	75	63	33	Total litter weight born alive (28); Hippo pathway to control organismal growth and development (57)
*SLC36A4*	9	DSR2038	4	4	0	Litter weight born alive in pigs (42)
*PCDHAC2*	2	DSR447-466	239	187	35	Neural signal transduction in animals (58)
*PSD3*	17	DSR3462	8	6	0	Total litter weight (43) in pigs

CCDC85C, coiled-coil domain containing 85C; EFNA5, ephrin A5; FTO, FTO alpha-ketoglutarate-dependent dioxygenase; GPC6, glypican 6; PALLD, palladin, cytoskeletal-associated protein; PCDHAC2, protocadherin alpha subfamily C, 2; RFTN1, raftlin, lipid raft linker 1; STK3, serine/threonine kinase 3; SLC36A4, solute carrier family 36 member 4; PSD3, pleckstrin and Sec7 domain containing 3.

Between WC and WZS pigs, 3,530 DSRs containing 594 unique genes were identified in the WZS and WC pigs (Supplementary Table S4). Many candidate genes were found to be associated with economically important traits. For example, *GPC6*, *GRM4*, and *VWA8* were related to body growth, while *ESRRG* and *ENOX1* were linked to reproductive ability (Table 3 and Figure 3C). Moreover, genes harboring SNPs with extremely high *F_ST_* values, such as *DSCAM*, *ADAMTS14*, and *AGXT2*, were identified.

**Table 3:** Candidate genes associated with important traits between WC and WZS.

Gene	Chr	DSRs	Number of SNPs	Top 0.5%	Top 0.01%	Function
*ESRRG*	10	DSR2101-2104	37	30	0	Placental physiology (38); Litter size (39)
*ENOX1*	11	DSR2325	3	3	2	Number born alive (28); Backfat thickness (28); Litter variability in pigs (41)
*GLG1*	6	DSR1181-1191	228	188	47	Biosynthesis of FSH and LH (59)
*GPC6*	11	DSR2408-2418	90	77	1	Skeletal growth in mice (35)
*GRM4*	7	DSR1376-1378	38	31	7	Backfat thickness (28); Loin muscle area (28); Body size in pigs (36)
*STK3*	4	DSR738-741	31	21	9	Hippo pathway to control organismal growth and development (57)
*VWA8*	11	DSR2329-2331	30	24	2	ADG (28); Body size in pigs (37)

ENOX1, ecto-NOX disulfide-thiol exchanger 1; ESRRG, estrogen-related receptor gamma; GLG1, Golgi glycoprotein 1; GPC6, glypican 6; GRM4, Glutamate metabotropic receptor 4; STK3, serine/threonine kinase 3; VWA8, Von Willebrand factor A domain containing 8; ADG, average daily again.

## 4 Discussion

Historically, the local pig breeds on Hainan Island, including DT, WZS, and WC pigs, have distinct genetic backgrounds. As mentioned above, DT pigs are a breed that was developed as a result of residents introducing pig breeds from Guangxi, Guangdong, and Vietnam before 1949, followed by a long-term process of selective breeding (1, 2), while WZS pig breed originates from the mountainous regions of Wuzhishan city in central Hainan and has adapted to the local climate and geographical conditions. Additionally, the WC pig breed was introduced from southern China. It originated from Gongguan pigs brought in by residents from the Guangdong coast during human migrations that took place around AD 1600–1700 (1). These histories indicate that DT, WZS, and WC represent the three origins of Hainan pig breeds: DT pigs originated from hybridizing foreign and local pigs, WZS pigs are local pigs, and WC pigs are introduced to foreign pig breeds. In this study, data from the neighbor-joining tree and genetic relationship plots indicated that the pig breeds formed four distinct clusters. Genetic differentiation analysis found that the *F_ST_* value between DT and WZS pigs was only 0.09, while the *F_ST_* values for WC were higher than those of the other two breeds (0.17 between WC and DT, and 0.17 between WC and WZS), indicating that DT pigs have a closer genetic relationship with WZS pigs. According to Wright’s original description of *F_ST_*, it is generally accepted that when the value of *F_ST_* ranges from 0.05 to 0.15, there exists a moderate level of genetic differentiation between the two populations. Conversely, when the *F_ST_* value falls within the range of 0.15–0.25, a relatively high degree of genetic differentiation is considered to exist between the two populations (29). With reference to this criterion, our findings demonstrate that a relatively high degree of genetic differentiation has already emerged between WC and the other two pig breeds. In contrast, a moderate level of genetic differentiation exists between DT and WZS pigs. Our results provide firsthand genome-wide data for discovering the genetic relationships among three local Hainan pig breeds with different geographical and historical origins.

As the only breed among the three pig breeds that originated locally, WZS pigs are a small breed in China with slow growth, weighing only 30 kg at 30 months of age. In contrast, the DT and WC pigs are medium or large-sized breeds, with adults weighting around 60–70 kg for DT boars and 120 kg for WC boars at 24 months of age (1). This shows a huge difference in growth performance between WZS and the other two pig breeds. As expected, many key genes known to influence growth, skeletal and muscle development, and body size, such as *GHR*, *ARL15*, and *PLD1* between WZS and DT pigs, and *GPC6*, *GRM4*, and *VWA8* between WZS and WC pigs, were identified in this study. GHR plays a crucial role in the GH-GHR-IGF1 axis by transmitting growth hormone signals to cells and regulating IGF1 expression, thereby controlling growth and metabolism (30). Mutations in *GHR* result in dwarfism in humans and animals (31), and the deletion of *GHR* creates dwarfism models in miniature pigs (32). *ARL15* and *PLD1* have been reported as candidate genes for feed conversion efficiency or body growth, and SNPs in *ARL15* are considered important QTL markers influencing the age at 100 kg of pigs (33, 34). Additionally, *GPC6* promotes skeletal muscle development through the Hedgehog signaling pathway (35), whereas mutations in *GRM4* and *VWA8* are related to pig body size (36, 37). These data provide strong evidence for the differences in growth performance between WZS and DT or WC pigs.

Among the local pig breeds in Hainan, WC pigs have a better litter size and are often used for hybridization with Duroc pigs to produce black pigs, whereas the litter size of WZS pigs is extremely poor, with almost half that of WC pigs (1). In the genome, we identified several DSRs between WZS and WC pigs that involved genes related to reproductive ability, such as *ESRRG* and *ENOX1*. *ESRRG* is an estrogen-related receptor that plays an important role in the physiological function of the placenta and maintenance of pregnancy (38). Mutation in *ESRRG* are associated with litter size in Landrace pigs (39) and can leads to placental dysfunction, which in turn affects reproduction; *ENOX1* plays a key role in cell growth (40), and its polymorphisms were shown to correlate with litter size in large white pigs (41), demonstrating that these two genes may play an important role in the farrowing or reproductive ability of pigs. More evidence is required to confirm whether the *ESRRG* and *ENOX1* genes are associated with the differences in reproductive performance between WC and WZS pigs.

Compared to WC pigs, DT pigs have a higher litter weight and are often used to produce roasted suckling pigs. Some DSRs located in genes associated with litter weight, such as *SLC36A4* and *PSD3*, were also identified. *SLC36A4* is located on a QTL associated with the live weight of pig litter (42), whereas *PSD3* is associated with the total litter weight of pigs (43). These two genes may explain the unique differences in litter weights between DT and WC pigs. In addition, genes related to fat development and meat quality were identified in the DSRs. *FTO* for fat development and *EFNA5* and *RFTN1* for meat quality. *FTO* is a known key gene associated with obesity in humans through the GWAS study (44) and fat deposition on various animals, including pigs (45) and cattle (46), whereas *EFNA5* and *RFTN1* are associated with meat color (47, 48). Adult WC pigs exhibit an intramuscular fat content of more than 3%, which is significantly higher than the approximately 2.2% of DT pigs (1), implying that WC pigs may have better meat quality. Therefore, these three genes may contribute to the differences in meat quality between DT and WC pigs. However, more studies are needed to confirm the mechanism by which these genes affect the meat quality of pigs.

## 5 Conclusion

In summary, this study identified genetic relationships and numerous DSRs among the three Hainan pig breeds, which correspond to many genes potentially influencing the germplasm characteristics of these pig breeds, such as *GHR* between DT and WZS, *FTO* between DT and WC, and *STK3* between WC and WZS. These data provide a new reference basis for the relationship among pig breeds of different geographical origins and insight into the genome differences contributing to the unique traits of Hainan pig breeds.

## Data Availability

The datasets presented in this study are deposited in the China National Center of Bioinformation, accession number PRJCA032186, https://ngdc.cncb.ac.cn/bioproject/browse/PRJCA032186.

## Footnotes

^1^ https://www.ebi.ac.uk/

^2^ https://ftp.ensembl.org/pub/release-109/gff3/sus_scrofa/
